# Metabolic Control of Cell Elongation and Cell Division in *Bacillus subtilis*

**DOI:** 10.3389/fmicb.2021.697930

**Published:** 2021-06-25

**Authors:** Anne Galinier, Elodie Foulquier, Frédérique Pompeo

**Affiliations:** Laboratoire de Chimie Bactérienne, UMR 7283, CNRS/Aix-Marseille Université, Institut de Microbiologie de la Méditerranée, Marseille, France

**Keywords:** *Bacillus subtilis*, metabolism, cell elongation, MreB, cell division, FtsZ

## Abstract

To survive and adapt to changing nutritional conditions, bacteria must rapidly modulate cell cycle processes, such as doubling time or cell size. Recent data have revealed that cellular metabolism is a central regulator of bacterial cell cycle. Indeed, proteins that can sense precursors or metabolites or enzymes, in addition to their enzymatic activities involved in metabolism, were shown to directly control cell cycle processes in response to changes in nutrient levels. Here we focus on cell elongation and cell division in the Gram-positive rod-shaped bacterium *Bacillus subtilis* and we report evidences linking these two cellular processes to environmental nutritional availability and thus metabolic cellular status.

## Introduction

To survive in their environment, bacteria have to rapidly adapt to nutrient availability. For example, in rich nutritional conditions, bacterial size can be increased and doubling time can be notably reduced in comparison to poor growth conditions (Schaechter et al., 1958; Sargent, 1975). This observation suggests that cell cycle processes like cell length doubling, duplication then separation of DNA, and finally cell division are highly linked to central metabolism to ensure a viable progeny (Sperber and Herman, 2017).

*Bacillus subtilis* is the best-characterized Gram-positive endospore-forming bacteria. This aerobic rod-shaped bacterium belongs to the Firmicute phylum. It grows by elongation along its long axis until cell length has doubled and then divides at midcell to give two identical daughter cells.

**FIGURE 1:**
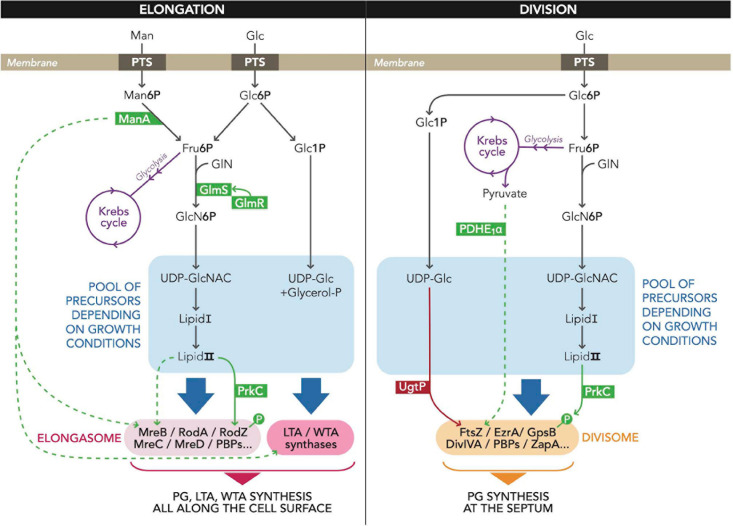
Schematic summary of metabolic regulations of CW elongation and cell division in *B. subtilis* described in the review. The two panels represent regulations of bacterial CW elongation (left) and cell division (right) by proteins whose activity or function is modulated by metabolic intermediates. Metabolic changes adjust the pool of precursors, visualized in blue boxes, in order to regulate LTA, WTA, or PG synthesis. Metabolic reactions are drawn with black arrows and the enzymes involved in the regulations are represented in green or red boxes. The several levels of regulation are mapped by dotted arrows, green for activations and red for inhibitions. When direct interaction exists between proteins and metabolites, dashed arrows are converted to solid lines. The available precursors are then used by LTA synthases and proteins from the elongasome or the divisome for CW synthesis all along the cell surface or at the septum, respectively. Abbreviations: Man, mannose; Man6P, D-mannose 6-phosphate; Glc, glucose; Glc6P, D-glucose 6-phosphate; Glc1P, D-glucose 1-phosphate; Fru6P, fructose 6-phosphate; GlN, glutamine; GlcN6P; glucosamine-6-phosphate; UDP-GlcNAc, uridine diphosphate N-acetylglucosamine; UDP-Glc, uridine diphosphate glucose; AcylACP, acyl-acyl carrier protein; lipid I, undecaprenyl-phosphate UDP-MurNAc-pentapeptide; lipid II, undecaprenyl-phosphate UDP-GlcNAc UDP-MurNAc-pentapeptide; PG, peptidoglycan; WTA, wall teichoic acids; LTA, lipoteichoic acids; PBP, penicillin-binding protein.

To grow, *B. subtilis* must synthetize the cell wall (CW) and the membrane. The main component of CW is the peptidoglycan (PG) in almost all bacteria (Egan et al., 2020). PG is a three-dimensional polymer that surrounds the surface of the cell and that is continuously remodeled during growth. It is composed of glycan chains crosslinked by short peptides and covers all the cytoplasmic or inner membrane. PG precursors like uridine diphosphate N-acetylglucosamine (UDP-GlcNAc) and lipid II are synthesized in the cytoplasm (Mengin-Lecreulx et al., 1982; van Heijenoort, 2007). Then, lipid II, a disaccharide pentapeptide coupled to bactoprenol, is exported to be integrated into existing CW sacculus (Mengin-Lecreulx et al., 1982; van Heijenoort, 2007). More precisely, lipid II is flipped outside the cell and assembled into the existing sacculus by penicillin-binding proteins (PBPs), enzymes that possess transglycosylases (TG) and transpeptidase (TP) activities. These enzymes are required to extend the glycan strands and construct peptide cross bridges (Errington and Wu, 2017; Egan et al., 2020). These PBPs are well characterized and are the targets of several antibiotics (Hugonnet et al., 2016; Sharifzadeh et al., 2020). They act in collaboration with RodA and FtsW, SEDS-family TG, to enable, respectively, elongation and division of the bacterial cell (Meeske et al., 2016; Emami et al., 2017; Sjodt et al., 2020). Extracellular enzymes with autolytic activities are also necessary to permit the expansion of the PG by breaking bonds in pre-existing material. All these enzymatic activities should be regulated to allow controlled elongation of the CW during growth (Sassine et al., 2020). Being just outside the cytoplasmic membrane, PG serves as a protective layer that protects the membrane against the turgor pressure due to the high osmolarity of the cytoplasm. The PG, with the help of the cytoskeletal proteins of the MreB family that guide PG synthesis and hydrolysis, contributes to the shape of the cell (Jones et al., 2001; den Blaauwen et al., 2008). MreB proteins are related to eukaryotic actins from a structural and biochemical point of view; they reversibly polymerize depending on the binding and hydrolysis of ATP (van den Ent et al., 2001b; Esue et al., 2005). *B. subtilis* possesses three actin-like proteins, MreB, Mbl, and MreBH, that possess some functional redundancies (Kawai et al., 2009). Deletions of *mreB* genes abolish rod-cell shape but overproduction of an MreB-like protein can compensate the absence of another (Kawai et al., 2009). Concerning the molecular role of MreB, it was proposed that this protein polymerizes as dynamic patches and orchestrates the elongasome (Domínguez-Escobar et al., 2011; Garner et al., 2011). In addition, it was recently shown that MreBH is essential for the activation of the major autolysin LytE and for its localization to the sites where the new PG is inserted (Carballido-López et al., 2006; Patel et al., 2020). The elongasome seems to be present only in rod-shaped bacteria, even if this point of view is debated, and directs lateral insertion of PG along the long axis of the cell to allow cylindrical growth (Szwedziak and Löwe, 2013). It is composed of a variety of enzymes (Figure 1), including MreB, MreC, MreD, RodA, RodZ, PBP2a, and PBPH (Errington, 2015; Errington and Wu, 2017; Egan et al., 2020; Patel et al., 2020).

The second major class of polymers that constitutes the bacillus CW are the teichoic acids (TAs) (Auer and Weibel, 2017; Wu et al., 2020). In many Gram-positive bacteria, there are two main forms, the wall TAs (WTA) covalently linked to the PG and lipoteichoic acids (LTA) connected to a lipid carrier. In *B. subtilis*, these two TAs have the same global composition and are mainly constituted of poly-[glycerol-phosphate].

*Bacillus subtilis* grows by cylindrical elongation and divides at the middle of the cell. This process necessitates a reorientation of CW synthesis. This process is orchestrated by a tubulin-like protein, FtsZ. This cytoskeletal protein is present in almost all bacteria. It is structurally and biochemically related to eukaryotic tubulin and its reversible polymerization is regulated by binding and hydrolysis of GTP (van den Ent et al., 2001a); it governs cell division (Aldea et al., 1990). Actually, FtsZ polymerizes to form a circumferential ring (also called Z-ring) that defines the site of cell division (or septum) (de Boer et al., 1992; RayChaudhuri and Park, 1992). In addition, FtsZ also recruits several proteins (more than a dozen of proteins identified in *B. subtilis*) involved in the division machine, also called the divisome (Gamba et al., 2009; Errington and Wu, 2017). The presence of a divisome is almost ubiquitous but its composition varies depending on bacteria; it is responsible of the constriction of inner and outer membranes and the PG synthesis at the division site (Szwedziak and Löwe, 2013). In *B. subtilis*, it was observed that the assembly of divisome occurs in two steps (Gamba et al., 2009). In the first step, Z-ring assembles early and concomitantly with FtsA, ZapA, and EzrA. Then, a second set of division proteins, including GpsB, FtsL, DivIB, FtsW, PBP2b, and DivIVA, are recruited to the middle of the cell. Several regulators of the divisome function have been identified; they are mostly inhibitors of FtsZ (Errington and Wu, 2017).

The aim of this mini review is to focus on how the cellular metabolism regulates cell cycle. Indeed, to spatially and temporally regulate their cell cycle according to nutritional availability, bacteria possess proteins that bind metabolites or precursors or metabolic enzymes that, in addition to their dedicated enzymatic functions, participate in cell cycle regulation. In this review, we describe metabolic proteins that regulate cell elongation (and cell shape) and cell division depending on growth conditions in the Gram-positive rod-shaped bacterium *B. subtilis.*

## Metabolism and Regulation of Cell Elongation and Cell Shape

### Stimulation of PG Synthesis by GlmR During Growth on Non-glycolytic Carbon Sources

The GlmR protein (YvcK) is a good illustration of a link between carbon metabolism and cell elongation. This protein is present in many bacteria, but it has been mostly studied in *B. subtilis.* Although *glmR* is dispensable during growth on glucose or glycolytic carbon sources, cells with a *glmR* deletion have abnormal shape and lyse under non-glycolytic growth conditions, like growth in the presence of Krebs cycle intermediates and substrates of pentose phosphate pathway (Görke et al., 2005). GlmR overproduction was shown to compensate the absence of MreB and its localization was similar to that of MreB suggesting a putative role in CW synthesis or in cell elongation (Foulquier et al., 2011). It was demonstrated that GlmR binds to UDP-GlcNAc, a cytoplasmic precursor of PG synthesis (Foulquier and Galinier, 2017). However, mutations in GlmR affecting its binding to UDP-GlcNAc do not perturb bacterial growth on non-glycolytic carbon sources. In a genetic study, it was proposed that GlmR stimulates the activity of GlmS (Figure 1) and the binding of UDP-GlcNAc to GlmR could prevent this stimulation (Patel et al., 2018). GlmS is an enzyme involved in the synthesis of UDP-GlcNAc; it converts fructose 6-phosphate (Fru6P) and glutamine (GlN) into glucosamine-6-phosphate (GlcN6P) and glutamate (Badet et al., 1987). By measuring the production of GlcN6P with purified proteins, it was indeed shown that GlmR directly stimulates GlmS activity and this positive effect is inhibited by UDP-GlcNAc (Foulquier et al., 2020). Combining these genetic and biochemical studies, the role of GlmR was elucidated (Foulquier and Galinier, 2017; Patel et al., 2018; Foulquier et al., 2020). When growth conditions are poor (non-glycolytic carbon sources), GlmR is essential because the intracellular concentration of UDP-GlcNAc is low. GlmR needs to interact with GlmS to stimulate GlmS activity and thus permit a normal PG synthesis and an efficient CW elongation. Indeed, during gluconeogenesis since Fru6P, the substrate of GlmS is present at low levels. The GlmR-stimulatory effect is probably essential under these conditions. In rich growth conditions, the intracellular concentration of UDP-GlcNAc is high and GlmR binds to this UDP-sugar. GlmR does not interact with GlmS and thus does not stimulate its activity. A *glmR* deletion has thus no effect in such conditions (Patel et al., 2018; Foulquier et al., 2020).

### ManA: A Protein That Links Metabolism and Cell Shape

Phosphomannose isomerase (ManA) is another protein in *B. subtilis* that links metabolism and cell shape. ManA is a mannose phosphate isomerase that converts D-mannose 6-phosphate (Man6P) to D-Fru6P when bacteria are grown on mannose as carbon source (Sun and Altenbuchner, 2010). However, the role of this enzyme is not limited to mannose utilization. Indeed, ManA is also necessary for normal rod shape when bacteria are grown in LB medium that does not contain mannose. In such growth conditions, the presence of ManA avoids chromosome polyploidy and abnormal morphologies (Elbaz and Ben-Yehuda, 2010). This observation was completely unexpected and mysterious. It is noticeable that the deletion of *pmi*, the gene encoding a second mannose phosphate isomerase, had no observable phenotype when bacteria were grown in LB medium, indicating a specific role of ManA. The authors observed that the CW of the *manA* mutant composition has a significant decrease in the amounts of four carbohydrates that are also typical components of WTA. In contrast, the concentration of GlcNAc, the major PG carbohydrate, was similar in wild-type and Δ*manA* cells. Thus, the authors proposed that ManA is required for proper formation of WTA (Figure 1) and thus to maintain an equilibrium between PG and WTA; this proper balance is crucial for a correct CW formation. As a result, the deletion of *manA* causes an abnormal CW synthesis that induces a deregulation and an asynchrony between cell elongation, division, and nucleoid segregation (Elbaz and Ben-Yehuda, 2010). Up to now, the molecular mechanism in which ManA is implied is uncharacterized.

### Regulation of MreB Dynamics by the Richness of the Growth Medium

Other connections were observed between MreB and richness of growth conditions. Indeed, during fast growth in rich medium, bacteria need a faster synthesis of PG in comparison to slow growth in poor medium. For example, to adapt to nutrient shifts, while *Escherichia coli* potentially regulates the amount of PG inserted by MreB patches, *B. subtilis* regulates the dynamics of MreB patches (Billaudeau et al., 2017). Indeed, it was shown that the speed of MreB patches is correlated to the growth rate in *B. subtilis* and the richness of the growth medium, with a cell elongation per patch that is constant. More the medium is rich in nutrients, more the dynamics of MreB patches is high. Another recent study demonstrates that the membrane fluidity and thus MreB dynamics depend on growth conditions (Zielińska et al., 2020). This regulation is mediated by membrane proteins that form microdomains, the flotillins. These proteins may recruit specific lipids more or less rigid and whose synthesis could be dependent on the nutrient content of the growth medium. The authors observed that the addition of fluidizer is able to restore MreB dynamics and a normal cell shape to fast growing *B. subtilis* mutant cells that lack flotillins. They propose that, in rich medium when the bacteria are growing fast, the control of the membrane fluidity by flotillins, and thus the dynamics of MreB patches, is essential and sufficient for an efficient PG synthesis (Zielińska et al., 2020).

### Regulation of MreB by Lipid II

Other studies demonstrate an additional level of regulation of MreB via the PG precursor lipid II (Figure 1) (Schirner et al., 2015). Indeed, MreB membrane association was shown to be actively regulated and to depend on the presence of cytoplasmic lipid-linked PG precursors in *B. subtilis*. When these PG precursors are depleted (like in poor nutrient medium), MreB patches disassemble and PG synthesis becomes anarchic. In addition, dynamics of MreB patches are still observed in mutant cells that lack WTA but synthesize PG. However, these patches alone cannot maintain a cellular rod shape. The authors proposed that the cell regulates the interaction of the MreB patches with the membrane and this level of regulation allows a rapid and reversible inactivation of CW enzyme complexes in response to the inhibition of CW synthesis (Schirner et al., 2015). This also suggests that the availability of precursors, and in particular lipid II, at specific sites in the cell envelope likely acts upstream of MreB, in the spatial and temporal control of CW growth (Sperber and Herman, 2017). In complement to these data, a new study has very recently revealed another action of lipid II in the regulation of MreB via the serine/threonine kinase PrkC and the morphogenic protein RodZ (Sun and Garner, 2020). Indeed, comparing the single-cell growth rate to the density of moving MreB patches under different conditions, it was observed that the density of MreB patches is related to the growth rate. This effect requires the *mur* genes involved in biosynthesis of lipid II. In stationary phase, during germination and probably in poor growth conditions, the Ser/Thr kinase PrkC interacts with the lipid II or PG fragments via its extracellular domain (Shah et al., 2008; Squeglia et al., 2011; Pompeo et al., 2018). This interaction induces the oligomerization of PrkC that is evenly distributed in the CW and stimulates its kinase activity (Pompeo et al., 2018). During exponential growth in nutrient-rich conditions, PrkC has a septal localization. In such conditions, its activity is not regulated via its extracellular domain and interaction with PG fragments or lipid II but it requires the division protein GpsB (Pompeo et al., 2015, 2018). PrkC has several substrates and probably connects CW state to cellular processes. In particular, it phosphorylates GlmR to regulate its activity (Foulquier et al., 2014) and also the morphogenic protein RodZ (Ravikumar et al., 2014) (Figure 1). Phosphorylated RodZ increases the density of MreB patches (Sun and Garner, 2020). As a result, the cell elongation is stimulated and the growth rate increases. In addition, an overproduction of PrkC results in cells that elongate faster than wild type in nutrient-poor conditions. The authors propose that PrkC may act as a cellular rheostat, adapting cellular processes in response to lipid II (and also probably to PG fragments), allowing cells to adjust their growth under various nutrient conditions.

## Metabolism and Cell Division

### Regulation of Z-Ring Formation by UDP-Glucose and UgtP

A link between richness of growth medium and cell division was highlighted showing a direct regulation of FtsZ polymerization by a metabolic enzyme, UgtP, in *B. subtilis* (Weart et al., 2007). This protein acts a key metabolic regulator of cell division and is involved in glucolipids synthesis; it transfers Glc from UDP-Glc to diacylglycerol-containing sugar acceptors. Deletion of the *ugtP* gene induces smaller cells than wild-type cells. In fact, UgtP binds to FtsZ (Figure 1) and inhibits its polymerization in a UDP-Glc-dependent manner both *in vivo* and *in vitro* (Weart et al., 2007; Chien et al., 2012). In addition, it was shown that UDP-Glc, and consequently nutrient availability, modulates UgtP levels and localization. In summary, under rich-nutrient conditions, when UDP-Glc and UgtP intracellular concentrations are high, UgtP is localized to the septum where it binds to FtsZ to inhibit Z-ring formation. Cell division is thus delayed until the cell reaches their critical mass. Once critical mass is attained, it was proposed that a second unknown nutrient-dependent sensor alleviates this inhibition of cell division and permits progression through the remainder of the division cycle (Weart et al., 2007). In such conditions, cells are long. In poor-nutrient medium, when UDP-Glc level is low, UgtP intracellular concentration is reduced and the protein is sequestered in randomly positioned foci. In such conditions, UgtP does not inhibit FtsZ polymerization and cell division occurs. Thus, the changes in UgtP levels, that depend on nutrients and are coupled with a substrate-dependent localization, link cell division with growth rate in *B. subtilis* (Weart et al., 2007).

### Regulation of Cell Division by Pyruvate and Glycolysis

Another level of regulation of cell division by carbon metabolism was also emphasized in *B. subtilis*; this regulation involves glycolysis enzymes and production of pyruvate (Monahan et al., 2014). Indeed, *B. subtilis* strain deleted for *pyk*, the gene encoding the pyruvate kinase, an enzyme that produces pyruvate from phosphoenolpyruvate (PEP) in the final reaction of glycolysis, exhibits aberrant division events at the cell poles. The mutant cells have defects in FtsZ assembly with mislocalized Z rings. Addition of exogenous pyruvate reestablishes a normal cell division in these mutants. This observation supports the idea that pyruvate is a metabolite that plays an essential role in the coordination of bacterial cell cycle with cellular metabolic status. It was proposed that the pyruvate level is correlated to Z-ring polymerization via the E1α subunit of pyruvate dehydrogenase (PDH E1α), an enzyme that metabolizes pyruvate (Figure 1). Importantly, this enzyme localizes in a pyruvate-dependent manner over the nucleoid (Monahan et al., 2014). In this model, PDH E1α may be involved in the stimulation of Z ring formation at midcell when bacteria are cultivated in nutrient-rich conditions. In such conditions, cells divide more often due to a shorter mass doubling time. The quantity of PDH E1α localized within this central region increases with the augmentation of nutrient levels; this could deliver a positive signal for Z ring formation that becomes stronger under rich conditions. However, up to now, it is not known how PDH E1α regulates FtsZ (directly or indirectly), how it affects cell division, and how it colocalizes with the nucleoid.

## Conclusion

In this mini review, we focused on the well-studied Gram-positive bacterium *B. subtilis* and showed some examples of how this bacterium adapts its cell division and elongation in response to its metabolism (Figure 1). The understanding of this adaptation is central in microbiology. Recent findings showed that metabolic enzymes play an essential role in this process. Indeed, in addition to their catalytic functions, these proteins, able to bind metabolites or precursors like UDP-Glc, UDP-GlcNAc, or Lipid II, sense the metabolic status of the bacterial cell. Then they transmit this information directly to the cell-cycle machineries, interacting with the elongasome, in particular with MreB, and/or with the divisome, in particular with FtsZ but not only. The discovery and the study of all those regulatory interactions that must occur to coordinate cell growth and division are a major focus in the future.

## Author Contributions

AG wrote the original draft. FP conceived Figure 1. AG, EF, and FP edited the manuscript. All authors contributed to the article and approved the submitted version.

## Conflict of Interest

The authors declare that the research was conducted in the absence of any commercial or financial relationships that could be construed as a potential conflict of interest.

## Abbreviations

Fru6P: fructose 6-phosphate
Glc: glucose
GlcN6P: glucosamine-6-phosphate
GlN: glutamine
LTA: lipoteichoic acids
Man6P: D-mannose 6-phosphate
PG: peptidoglycan
TA: teichoic acid
UDP-GlcNAc: uridine diphosphate N-acetylglucosamine
WTA: wall teichoic acids.
